# Recurrent *Campylobacter jejuni* Infection in an Immunodeficient Patient Treated with Repeated Faecal Microbiota Transplant (FMT)—A Case Report

**DOI:** 10.3390/idr14010007

**Published:** 2022-01-12

**Authors:** Blair Merrick, Aravind Gokul Tamilarasan, Raphael Luber, Patrick F. K. Yong, Kuldeep Cheent, Peter M. Irving, Manjula Meda, Simon D. Goldenberg

**Affiliations:** 1 Centre for Clinical Infection and Diagnostics Research, Department of Infectious Diseases, Guy’s and St Thomas’ NHS Foundation Trust and King’ College, London SE1 7EH, UK; blair.merrick@gstt.nhs.uk; 2 Department of Gastroenterology, Guy’s and St Thomas’ NHS Foundation Trust, London SE1 7EH, UK; gt.tamilarasan@hotmail.com (A.G.T.); raphaelluber@gmail.com (R.L.); peter.irving@gstt.nhs.uk (P.M.I.); 3 Department of Immunology, Frimley Health NHS Foundation Trust, Frimley GU16 7UJ, UK; patrick.yong@nhs.net; 4 Department of Gastroenterology, Frimley Health NHS Foundation Trust, Frimley GU16 7UJ, UK; kuldeep.cheent@nhs.net; 5 Department of Infection and Immunity, Frimley Health NHS Foundation Trust, Frimley GU16 7UJ, UK; m.meda@nhs.net

**Keywords:** *Campylobacter jejuni*, faecal microbiota transplant, common variable immunodeficiency, microbiota

## Abstract

There is limited evidence to guide successful treatment of recurrent *Campylobacter* infection in patients with common variable immunodeficiency (CVID) already managed on regular immunoglobulin therapy. The role of faecal microbiota transplant (FMT) is uncertain. We report a case of recurrent *Campylobacter jejuni* infection in a patient with CVID treated with repeated FMT with 18 months of symptom resolution prior to relapse.

## 1. Case Report

A 70-year-old female (b. 1951) with common variable immunodeficiency (CVID) since 2000 was first diagnosed with *Campylobacter jejuni* enterocolitis in 2012 (macrolide sensitive but quinolone and tetracycline resistant) following travel to Brazil. Additional infection-related complications of her CVID included Epstein–Barr virus-positive diffuse large B-cell lymphoma (DLBCL), *Toxoplasma*-related chorioretinitis, and chronic sinusitis with recurrent epistaxis. Other medical history included haemoglobin H (HbH) disease, cirrhosis complicated by oesophageal varices, pericarditis causing tamponade, a splenic artery aneurysm (identified on a routine CT and under regular review), ureteric stone treated with lithotripsy, and hypertension. Medication included intravenous immunoglobulin (IVIg) infusions (20 g 5% Flebogamma DIF every 2 weeks), prophylactic (thrice-weekly) trimethoprim–sulfamethoxazole, lansoprazole, losartan, and vitamin D.

Her initial *C. jejuni* infection was treated with a 7-day course of erythromycin with good clinical response. However, in 2014, when she developed another diarrhoeal illness, *C. jejuni* was again cultured. This recurrence coincided with two missed immunoglobulin infusions. She responded to a course of clarithromycin, to which the organism was sensitive. She remained asymptomatic until developing profuse diarrhoea in May 2018. *C. jejuni* was identified again, by polymerase chain reaction (PCR) and culture with the same resistance pattern as previously. She was treated with a 3-week course of clarithromycin, and her stool frequency improved to 3 times daily, albeit with regular loperamide use. Following this in August 2018, stool *C. jejuni* DNA was not detected. However, in December 2018, she again experienced a recurrence of profuse, non-bloody, watery diarrhoea, and *C. jejuni* was identified by PCR and culture (sensitivities as before). Despite a prolonged course (3 months) of clarithromycin, and an initial brief period of improvement, her symptoms persisted, and *C. jejuni* DNA continued to be detected on repeat stool testing. Culture undertaken in February 2019 demonstrated the organism had developed macrolide resistance (in addition to ciprofloxacin and tetracyclines).

At this point, due to lack of improvement and inability to eradicate *C. jejuni* with antibiotic therapy alone, faecal microbiota transplant (FMT) was considered. FMT delivery was preceded by a course of fosfomycin 3 g every 48 h for 2 weeks (given based on susceptibility testing and literature reports of success [1]). Then, 200 mL of frozen-thawed FMT derived from > 50 g of screened healthy donor faeces, manufactured as previously described [2] and in accordance with National guidelines [3], was delivered via push enteroscopy into the proximal jejunum after standard bowel preparation in early May 2019. The patient was discharged the same day and experienced clinical improvement within 48 h. However, although she gained a significant amount of weight, her bowel symptoms deteriorated, and *C. jejuni* was again identified (by both PCR (twice) and culture (once)) in late May 2019. The patient’s IVIg dose was temporarily increased to 15 g 5% Flebogamma weekly and she had a 2-week course of meropenem and then a 6-week course of fosfomycin, followed by a second FMT (identical protocol but using a different donor) delivered at the end of July 2019. Her stool tested negative (twice) by PCR for *C. jejuni* DNA in June 2019. Following this course of treatment, her symptoms markedly improved, and her weight continued to increase. Notably, when tested in August and November 2019, *C. jejuni* DNA was again detectable in stool by PCR.

She remained well until the latter part of 2020, when a new diagnosis of invasive ductal carcinoma of the breast was made in early December, for which she was commenced on hormonal therapy. She was admitted later that month with nausea, vomiting, and diarrhoea attributed to said therapy. Inflammatory markers were also raised, and she was additionally diagnosed and treated for left lower limb cellulitis with flucloxacillin. Upon discharge on 31 December 2020, she was reported to be clinically well with improved inflammatory markers. Admission blood cultures subsequently grew *C. jejuni* (this time macrolide sensitive), and stool had detectable *C. jejuni* DNA. By the time of the result, her clinical symptoms of diarrhoea had improved, but she was treated with a 7-day course of clarithromycin nevertheless. Her diarrhoea returned towards the end of this course of antibiotics on 6 January, by which time she had also developed a cough. She was seen by her GP and tested positive for SARS-CoV-2 on 10 January. She was admitted to the hospital later that day, where she deteriorated and died three days later. COVID-19 was listed as the primary cause of death, with CVID and HbH disease as contributory conditions. A summary of events is depicted in Table 1. 

## 2. Discussion

Common variable immunodeficiency (CVID) is the commonest symptomatic primary immunodeficiency. It is an umbrella diagnosis for a heterogeneous group of immune deficiency disorders that result predominantly in antibody deficiency, although cellular immune deficiency has been described. Multiple genetic anomalies have been demonstrated to result in a CVID-like clinical presentation, but they are only found in 10–20% of patients with CVID [4]. Intravenous immunoglobulin replacement has vastly altered the prognosis and severity of complications; however, infectious and non-infectious co-morbidities persist. In a cohort study in the Netherlands examining 49 patients with either X-linked agammaglobulinaemia (XLA) or CVID, *C. jejuni* was the second most common gastrointestinal infection identified. Interestingly, *Campylobacter* infection appears to be less common in patients with CVID now, compared with historical records, although the reason for this remains unclear. The purported rationale behind increased susceptibility to *Campylobacter* in CVID is that patients are deficient in IgA, a major part of the gastrointestinal tract’s defence against *C. jejuni*. Nevertheless, this cannot fully explain the increased propensity, as patients with selective IgA deficiency alone are not at significantly increased risk. In addition, CVID patients are liable to develop more serious complications, including bacteraemia, as they lack bactericidal activity derived from plasma IgM [5] and have increased intestinal permeability, making bacterial translocation more likely [6]. 

The clinical course of our patient paralleled the commonly described pattern of complications attributed to CVID. Apart from the infectious co-morbidities (recurrent *Campylobacter*, *Toxoplasma* chorioretinitis, and chronic sinusitis), previous DLBCL is also in keeping with CVID, with a reported 30-fold increase in lymphoma incidence [7].

*Campylobacter* species are amongst the most common enteropathogens in developed countries and a frequent cause of foodborne enteric infection. Typically, *Campylobacter* gastroenteritis results in a mild-to-moderate, usually self-limiting, infection that often does not require specific antimicrobial treatment. Where treatment is necessary, macrolides are presently the antimicrobial of choice. Fluoroquinolones are avoided unless susceptibility is confirmed, due to high resistance rates, and alarmingly, there are increasing reports of macrolide resistance, particularly in developing countries [8]. In the case presented, the drug sensitivity pattern mirrors this with initial fluoroquinolone resistance and later development of macrolide resistance. Given the recurrent infections with limited remissive intervals, similar resistance patterns, and identical typing (when performed at the reference laboratory in December 2018 and April 2019), it is presumed that the patient’s infection represented *Campylobacter* persistence/relapses rather than multiple re-infections. It is also presumed that the organism developed macrolide resistance in vivo, which has previously been reported [9].

The development of antimicrobial resistance has led to the use of older agents, such as fosfomycin, and broad-spectrum agents, such as meropenem. Success in treating *Campylobacter* enteritis with fosfomycin has been described [1,10], and meropenem has been recommended in the setting of outbreaks with multi-drug resistant isolates [10,11]; however, no clinical breakpoints exist for these agents. In our case, both were employed in an attempt to treat and eradicate any persistent *Campylobacter* reservoirs, followed by FMT, to restore a healthy colonic microbiota and reduce the risk of relapse. It is possible that the longer antimicrobial course or combination of antimicrobials (eight weeks of fosfomycin/meropenem prior to the second FMT vs. two weeks fosfomycin prior to the first FMT) contributed to the prolonged clinical response seen after the second FMT.

Faecal microbiota transplantation (FMT) involves the transfer of healthy donor faeces to a ‘diseased’ recipient with the primary purpose being to restore a ‘normal’ colonic microbiota. This has been shown to be a highly effective treatment strategy for recurrent and refractory *Clostridioides difficile* infection (CDI) [3]. It has also shown potential as a therapeutic option for mild to moderate ulcerative colitis to induce remission [12], to treat the dysbiosis associated with chronic liver disease [13], and for the eradication of gastrointestinal carriage of antibiotic-resistant organisms [14]. It has also been shown to reduce antibiotic-resistance genes in patients who underwent FMT for recurrent CDI [15]. There are cases in which FMT has been reported to successfully eradicate other bacterial pathogens, e.g., Salmonella (both chronic, asymptomatic carriage and active, symptomatic infection). In one study, two immunocompromised patients with symptomatic, persistent, non-typhoidal *Salmonella* (*Salmonella infantis*) infection, despite numerous courses of antibiotics, were treated successfully with a prolonged course of ertapenem, followed by encapsulated FMT [16]. It is difficult to say to what degree FMT contributed to cure, as both patients had absent or scant (normal) aerobic flora and no detectable *Salmonella* prior to FMT. Notably, one of the patients had similarities to our patient, having been diagnosed with hypogammaglobulinaemia secondary to chronic lymphocytic leukaemia (CLL), requiring IVIg [16]. In the second study, two patients with asymptomatic salmonella carriage were successfully treated with FMT, preceded by daily ceftriaxone [17]. However, to our knowledge, there are no published reports describing the use of FMT to treat *Campylobacter* infection, regardless of the outcome. 

Studies have reported differences in the composition of gut microbiota in those susceptible to infection with *Campylobacter*, with some specific genera being associated with resistance to colonisation on exposure [18]. Abattoir workers who remained *Campylobacter* negative had an overrepresentation of Clostridiales, Lacnospiraceae, and *Anaerovorax* species, and a lower abundance of *Bacteroides*, *Escherichia* and *Streptococcus* species. The same group identified similar findings in travellers, and additionally, that lower overall bacterial diversity leads to an increased risk of enteropathogen infection, including *Campylobacter* [19].

Emerging data suggest overall bacterial diversity is lower in individuals with CVID, compared with healthy controls [5]. There are also higher levels of plasma lipopolysaccharide and markers of immune activation (e.g., soluble CD14 and CD25), suggesting the breakdown of the gut barrier and disordered microbiota-mucosal immunity [20]. FMT is proposed to work (at least in the context of *C. difficile*) to increase bacterial diversity with an ensuing increase in short-chain fatty acid production and secondary bile acid production, restore gut barrier integrity, and modulate microbiota–mucosal immune interplay, including the production of antimicrobial peptides [21]. These alterations promote colonisation resistance to enteropathogens. However, if the described changes are intrinsic to CVID, and microbiota modulation with FMT does not (permanently) reverse some or all of these, then the ultimate inability to prevent colonisation and infection with *C. jejuni* in our patient, even with repeated FMT, is understandable. Furthermore, antimicrobial therapy is well recognised to perturb the intestinal microbiota. Our patient was taking long-term co-trimoxazole (for prophylaxis) and received numerous other courses of antibiotics as part of their ongoing care. The impact of these agents on intestinal microbiota may also have attenuated colonisation resistance.

The use of FMT derived from two different donors suggests that eventual symptomatic recurrence was not due to donor selection (donor-dependent success has been suggested for other conditions) [22] but cannot be fully excluded. We did not undertake serial microbiome or other analyses (e.g., metabolomics) in our patient, thus cannot further explore these hypotheses.

In summary, we report on a case of recurrent *C. jejuni* infection in a patient with CVID that was treated on two occasions with FMT with symptom resolution and clinical response following the second FMT, but with subsequent relapse after 18 months. The period of symptom resolution following the second FMT resolution considerably improved the patient’s quality of life, and thus, had they survived, a third FMT may have been considered. The understanding of the microbiota and its modulation in CVID is in its very infancy, and it is vital to report all outcomes, whether positive or negative, to expand the evidence base. 

## Figures and Tables

**Table 1:** Timeline of events from time of first episode of *Campylobacter* enteritis to death in January 2021.

Year	2012	2013	2014			2015	2016	2017	2018				2019														2020				2021
Month									M	A	S	D	J	F	M	A	M				J	J		A	S	N	J	M	J	D	J
Date																		8	23	31		30									
Antibiotic	Ery (1/52)				Clari (1/52)				Clari (3/52)			Clari (3/12)				Fos (2/52)				Mero (2/52)	Fos (6/52)									Fluclox (2/52)	Clari (1/52)
IVIg (g/ fortnight)	20	20	20	0	20	20	20	20	20	20	20	20	20	20	20	20	20	20	20	30	30	30	30	20	20	20	20	20	20	20	20
FMT																		1st					2nd								
*C.jejuni* (DNA)									+	ND (×2)	ND	+		+					+	+	ND (×2)			+		+				+	
*C.jejuni* (culture)	+ Stool				+ Stool				+ Stool			+ Stool		+ Stool						+ Stool										+ Blood	
Weight (kg)													42.6		45.2		44.4				48				48.8		51	50	49.2	47.1	

Ery = erythromycin, Clari = clarithromycin, Fos = fosfomycin, Fluclox = flucloxacillin, course length given in weeks (/52) or months (/12), + = detected, ND = not detected.

## Data Availability

Data is available on written request to the corresponding author.
